# Exploring the Effects of Cigarette Smoking on Inflammatory Bowel Disease Using Mendelian Randomization

**DOI:** 10.1093/crocol/otaa018

**Published:** 2020-03-12

**Authors:** Daniel P Jones, Tom G Richardson, George Davey Smith, David Gunnell, Marcus R Munafò, Robyn E Wootton

**Affiliations:** 1 Department of Population Health Sciences, Bristol Medical School, University of Bristol, Bristol, UK; 2 Southmead Hospital, Westbury-on-Trym, Bristol, UK; 3 MRC Integrative Epidemiology Unit, Bristol, UK; 4 NIHR Biomedical Research Centre, University Hospitals Bristol NHS Foundation Trust, University of Bristol, Bristol, UK; 5 UK Centre for Tobacco and Alcohol Studies, University of Bristol, Bristol, UK; 6 School of Psychological Science, University of Bristol, Bristol, UK

**Keywords:** smoking, inflammatory bowel disease, Mendelian randomization, UK Biobank

## Abstract

**Background:**

Previous observational evidence has suggested an association between smoking and inflammatory bowel disease (IBD).

**Methods:**

We used observational techniques followed by Mendelian randomization to explore whether smoking is a causal factor in the development of IBD and its subtypes.

**Results:**

In those who have ever smoked, we observed increased risk of IBD and, in current smokers, we observed increased risk of Crohn disease and decreased risk of ulcerative colitis. However, our Mendelian randomization analyses found little evidence that smoking affects the development of IBD.

**Conclusion:**

Overall, our results suggest that smoking does not causally influence the risk of IBD.

## INTRODUCTION

Inflammatory bowel disease (IBD) is a chronic condition affecting the gut. It presents in one of the two distinct clinical categories: Crohn disease and ulcerative colitis (UC). The prevalence of IBD in UK is 1% within the adult population and commonly manifests with chronic symptoms of abdominal pain, weight loss, and diarrhea as well as acute flares that can result in hospitalization, surgical interventions, and even death.^1^ It represents a substantial source of morbidity and is often difficult to manage medically.

Tobacco smoking is associated with both risk and clinical course of IBD. Ever smokers have an overall greater risk of developing IBD than never smokers^2^ (OR = 1.64 [95% CI 1.36–1.98] for UC, 1.80 [95% CI 1.33–2.51] for Crohn disease). The risk of developing IBD also differs between those who currently smoke and those who have previously smoked although this relationship varies depending on the clinical subtype. In a recent meta-analysis of 22 case–control studies, it was observed that current smokers have an increased risk of developing Crohn disease compared with former smokers (OR = 1.76 [95% CI 1.40–2.22]), whilst current smokers have a reduced risk of developing UC compared with former smokers^3^ (OR = 0.58 [95% CI 0.45–0.75]). This meta-analysis also highlighted a markedly increased risk of developing UC in the year following smoking cessation (OR = 1.79 [95% CI 1.37–2.34]).

Following disease onset, smoking in Crohn disease has also been associated with a worse clinical course, in terms of recurrence, need for surgical interventions, and disease complications.^4^ In contrast, the effect of smoking on the clinical course of UC appears to be protective in terms of recurrence, hospitalizations, and symptoms^5^ although there is some conflicting evidence.^6, 7^

The observational evidence certainly suggests an association between smoking and risk of IBD. There are several plausible mechanisms by which smoking could affect the etiology and course of IBD, including effects on the immune system and gut permeability.^8^ Tobacco smoke has also been shown to modify the association of certain genetic variants with risk of developing Crohn disease and UC.^9^ However, the observational evidence may well not represent true causation, given that observational data could be subject to bias from confounding (such as socioeconomic status or alcohol use) and reverse causation. One method that can be used to reduce bias from confounding and reverse causation is Mendelian randomization (MR).

**Figure 1.**
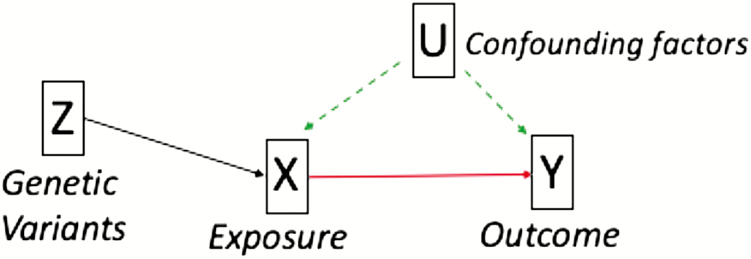
An illustration of MR principles where genetic variants (*Z*) are robustly associated with the exposure of interest (*X*) that in turn affects the outcome of interest (*Y*). These variants (*Z*) are not affected by confounding factors (*U*) or the outcome (*Y*) whereas the exposure alone (*X*) may be affected by both.

MR is an instrumental variable analysis that uses genetic variants as proxies for an exposure (eg, smoking) to estimate the causal effect on an outcome (eg, IBD). Genetic variants are randomly inherited at conception and should therefore be independent of confounding factors. As genetic variants are stable over the lifetime, the genetic instrument cannot be changed by the outcome, therefore, insuring there is no reverse causation^10^ (see Figure 1). MR can be conducted using measured genotypes, exposure, and outcome in a single sample, known as individual-level MR. Alternatively, summary-level MR can be conducted which uses genome-wide association study (GWAS) summary data from independent samples. This helps to significantly increase statistical power and avoids the cost and difficulty associated with measuring both the exposure and the outcome in the same sample. We used both MR methods to estimate the causal effect of smoking on risk of IBD to further elucidate the etiology of this complex disease.

In order to infer causality in MR, the genetic instrument must satisfy three assumptions: (1) the genetic variants must robustly predict the exposure of interest (with stronger instruments giving better power to detect effects), (2) the genetic variants must not predict confounding factors that affect the exposure and/or outcomes, and (3) the genetic variants must only affect the outcome via the exposure.^11^ Violation of these assumptions can occur in MR due to pleiotropy (where one genetic variant has multiple biological effects).^12^ We conducted multiple sensitivity analyses to ensure our results are reliable and not biased by pleiotropy.

In summary, we aimed to replicate previous observational associations between smoking and IBD subtypes. We followed up these associations using two MR methods to test whether there was evidence of smoking being a causal risk factor for IBD. Consistent effects triangulated across multiple MR methods would give the strongest evidence for a causal effect.^13^

## MATERIALS AND METHODS

### Observational Analysis

#### Sample

Analysis was conducted using the UK Biobank, a large national health resource aged 39–72 years at recruitment (mean 56.9 years [SD 8.00]). Over 500,000 participants were recruited between 2006 and 2010 from study centers across the UK. Further details can be found at www.ukbiobank.ac.uk. Individuals were excluded if they had withdrawn consent or if there were sex mismatches between reported and chromosomal sex or aneuploidy (*N* = 814). Individuals were restricted to European ancestry based on the first four principal components of population structure and related individuals were removed following MRC Integrative Epidemiology Unit filtering steps. The result was a sample of 337,053 individuals (54% female, 45% ever smokers (10% current; 35% former)).

#### Measures of IBD

Individuals were classified from hospital records as having a diagnosis of either Crohn disease or UC using ICD 10 codes K50 and K51 (field 41202). Individuals were classified as having IBD if they had a diagnosis of either UC or Crohn disease. For the full sample, 1.1% had any diagnosis of IBD, 0.4% had a diagnosis of Crohn disease, and 0.8% had a diagnosis of UC. When compared with the most recent study of a UK population, a similar 40- to 69-year-old cohort had prevalence of 1.1%, 0.4%, and 0.7% for IBD, Crohn disease, and UC, respectively.^14^

#### Statistical analysis

We looked at the effect of four smoking behaviors on IBD collectively and on each subtype. These were smoking status (ever vs never), smoking status (current vs former within ever smokers), cigarettes per day (within ever smokers), and lifetime smoking score. The latter is a combination of smoking duration, smoking cessation, and smoking heaviness described in detail elsewhere.^15^ The association between each of these smoking behaviors and IBD was estimated using logistic regression, controlling for age, sex, and socio-economic position (SEP). All analyses were conducted using R.^16^

### Individual-level MR Analysis

We conducted an individual-level MR analysis using a single single-nucleotide polymorphism (SNP) for smoking heaviness on IBD outcomes in the UK Biobank sample outlined above. We used the genetic variant rs1051730 (A/G) found in the gene cluster *CHRNA5-A3-B4.* This SNP is known to be associated with nicotine metabolism such that each allele increase in rs1051730 corresponds to an average of one more cigarette smoked per day.^17–20^

#### Genotype data

UK Biobank participants provided blood samples at the initial assessment center. Genotyping was performed using the Affymetrix UK BiLEVE Axiom array for 49,979 participants and using the Affymetrix UK Biobank Axiom array for 438,398 participants. The two arrays share 95% coverage, but the chip is adjusted for in all analyses because the UK BiLEVE sample is overrepresented for smokers. Imputation and initial quality control steps were performed by the Wellcome Trust Centre for Human Genetics resulting in over 90 million SNPs and indels. After excluding individuals who had withdrawn consent and using the most stringent ancestry exclusions, 337,053 of the participants remained.^20^ Participants’ genotype was categorized as 0,1, or 2 depending on the number of effect alleles (A).

#### Statistical analysis

We first checked the rs1051730 genotype was associated with cigarettes per day in smokers and split participants into ever smokers, current smokers, former smokers, and never smokers (as a negative control). Second, we checked that the genotype was not associated with demographic variables including age, sex, alcohol, and education. A logistic regression was run for genotype on each of the IBD outcomes adjusting for age and sex. Analysis was conducted in R, version 3.5.0.

### Summary-level MR

#### Instruments for the exposure

To construct our instruments for smoking exposure, we selected two smoking behaviors with known genetic proxies: smoking initiation and lifetime smoking. For smoking initiation, we used summary data from the GSCAN GWAS^21^ which defined smoking initiation as ever having smoked more than 100 cigarettes, smoking every day for at least a month or smoking regularly. The sample size was 1,232,091 and it identified 378 independent genome-wide significant genetic loci which explain 4% of the variance in smoking initiation. For lifetime smoking exposure, we used the “lifetime smoking index” which characterizes exposure as a combination of smoking initiation, duration, heaviness, and cessation. This index was originally based on data from a GWAS in the UK Biobank^13^ which identified 126 independent significant genetic loci in a population of 462,690. For SNPs present in our instruments but not our outcome summary data, we searched for suitable proxy SNPs from Phase 3 of the 1000 genomes project using the LDLink application,^22^ with an *R*^2^ of at least 0.8. Our final instrument SNPs have not previously been found to associate with IBD.

#### Outcome summary data

For the outcome of IBD, we used GWAS summary data from the most recent available GWAS.^23^ This study performed a case–control GWAS on 59,957 individuals of European Ancestry meta-analyzed across multiple cohorts. They identified 241 independent genome-wide significant SNPs, including 25 novel loci. Individuals were categorized according to the diagnosed histological subtype of IBD (UC and Crohn disease) as well as a combined IBD category which encompassed confirmed cases of UC, Crohn disease, and unclassified IBD.

#### Statistical Analyses

Analyses were conducted using the TwoSampleMR package 0.4.18^24^ in R version 3.5.1.^13^ We conducted the following MR methods: inverse-variance weighted (IVW),^25^ MR Egger SIMEX,^26^ weighted median,^27^ and weighted mode.^28^ Consistent effects across multiple methods that each make different assumptions about pleiotropy give us the strongest evidence for causal inference. The validity of the MR Egger method was evaluated using the regression dilution *I*^2^ statistic.^29^ If the result was less than 0.9, then SIMEX corrections were conducted. We assessed the strength of the genetic instruments using the Mean F-statistic. A value above 10 indicates good instrument strength.^30^ Bias from directional pleiotropy was specifically assessed using the MR Egger intercepts. We also applied Steiger filtering to remove the genetic variants that explain more variance in the outcome than the exposure^31^ as a test of reverse causation.

#### Data availability

The data used in the summary level MR analysis are publicly available via the cited references. The scripts used to conduct all analyses are available at https://github.com/MRCIEU/Smoking_IBD.

### Ethical Considerations

UK Biobank has received ethics approval from the UK National Health Service’s National Research Ethics Service (ref 11/NW/0382).

## RESULTS

### Observational Analysis

The prevalence in the UK Biobank was 9.9% for current smokers and 34.6% for former smokers. The prevalence of Crohn disease was 0.4% and UC was 0.8%. Observational analyses showed evidence for an association between smoking heaviness and risk of IBD but with small effect sizes (see Table 1). Smoking status (ever vs never) was associated with an increased risk of all IBD diagnoses whereas being a current smoker compared with a former smoker increased risk of Crohn disease and decreased the risk of UC (see Table 1).

**Table 1.** The Observed Association of Smoking Behavior and IBD Subtypes Controlling for Age, Sex, and Socio-Economic Position

	Inflammatory Bowel Disease			Crohn’s Disease			Ulcerative Colitis		
Smoking	*N*	OR (95% CI)	*P*-value	*N*	OR (95% CI)	*P*-value	*N*	OR (95% CI)	*P*-value
Ever vs Never	335,467	1.46 (1.37, 1.56)	<0.001	335,467	1.50 (1.34, 1.67)	<0.001	335,467	1.42 (1.31, 1.54)	<0.001
Current vs Former	151,645	0.78 (0.70, 0.88)	<0.001	151,645	1.24 (1.05, 1.47)	0.01	151,645	0.57 (0.49, 0.66)	<0.001
Cigarettes per day	100,029	1.01 (1.00, 1.01)	0.001	100,029	1.00 (0.99, 1.01)	0.40	100,029	1.01 (1.00, 1.02)	0.001
Lifetime smoking^a^	335,483	1.22 (1.17, 1.27)	<0.001	335,483	1.36 (1.27, 1.45)	<0.001	335,483	1.12 (1.06, 1.18)	<0.001

^a^A composite measure of smoking duration, intensity, and cessation. One unit increase in lifetime smoking is equivalent to having smoked 30 cigarettes a day for 17 years and stopping 13 years ago OR smoking 30 cigarettes a day for 19 years and stopping 15 years ago for example.

### Individual-level MR Analysis

**Figure 2.**
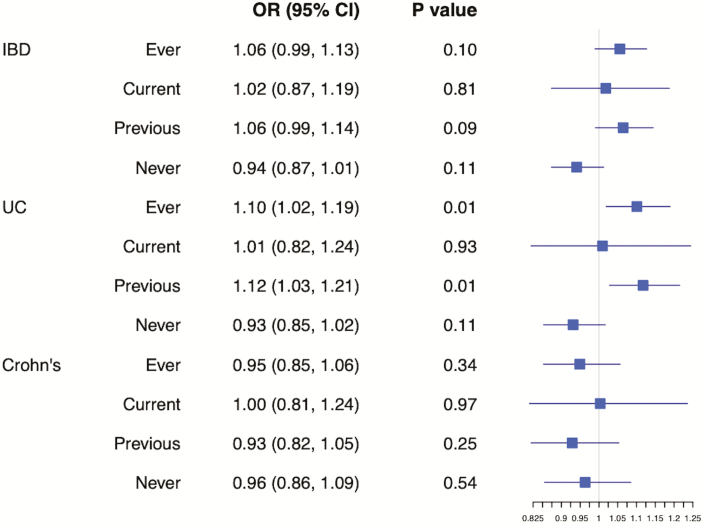
Single SNP analysis illustrating the effect of rs1051730 genotype (grouped by smoking status) on risk of IBD and its subtypes.

Each allele increase of rs1051730 genotype was associated with the expected increase of one more cigarette smoked per day on average among ever smokers (see Supplementary Table S1). In the single SNP analysis, strong evidence for a causal effect of smoking heaviness on IBD risk would be an effect in ever, current, or former smokers but no effect in never smokers. When looking at effects on IBD, there was only very weak evidence of rs1051730 effect alleles increasing the risk of IBD and UC in ever, current, and former smokers compared with never smokers. There was no evidence for an effect on Crohn disease (see Figure 2). Supplementary Table S5 shows the association of rs1051730 genotype with sample demographics.

### Summary-level MR Analysis

Of the 378 SNPs associated with smoking initiation, 325 were available (or had suitable proxies) in the GWAS summary statistics for IBD. Of the 126 SNPs associated with lifetime smoking, 107 were available (or had suitable proxies) in the GWAS summary statistics for IBD.

Overall, there was limited evidence for an effect of smoking on IBD. Our results showed weak evidence for smoking increasing the risk of developing Crohn disease (with notable effect sizes), but there was no clear evidence of an effect of smoking on the risk of IBD and UC. The confidence intervals for our results were wide and all crossed the null (see Table 2).

**Table 2.** Two-Sample Mendelian Randomization Analysis of the Effect of Smoking Behaviors on Risk of IBD and Its Subtypes

Exposure	Outcome	MR Method	OR (95% CI)	*P*-value
Smoking initiation	IBD	Inverse Variance Weighted	1.04 (0.93–1.17)	0.48
		MR Egger (SIMEX)	0.75 (0.41–1.38)	0.36
		Weighted Median	1.03 (0.91–1.18)	0.61
		Weighted Mode	0.98 (0.72–1.34)	0.92
	Crohn	Inverse Variance Weighted	1.14 (0.99–1.31)	0.07
		MR Egger (SIMEX)	0.78 (0.38–1.61)	0.51
		Weighted Median	1.09 (0.93–1.28)	0.30
		Weighted Mode	1.20 (0.74–1.96)	0.46
	UC	Inverse Variance Weighted	0.98 (0.86–1.11)	0.73
		MR Egger (SIMEX)	0.70 (0.36–1.37)	0.30
		Weighted Median	1.08 (0.92–1.27)	0.33
		Weighted Mode	1.22 (0.75–2.00)	0.42
Lifetime smoking	IBD	Inverse Variance Weighted	0.96 (0.72–1.27)	0.77
		Weighted Median	1.01 (0.76–1.34)	0.95
		Weighted Mode	1.11 (0.62–1.98)	0.72
	Crohn	Inverse Variance Weighted	1.31 (0.82–2.11)	0.26
		Weighted Median	1.63 (0.95–2.78)	0.07
		Weighted Mode	1.90 (0.54–6.73)	0.32
	UC	Inverse Variance Weighted	0.78 (0.58–1.05)	0.10
		Weighted Median	0.89 (0.62–1.28)	0.52
		Weighted Mode	1.34 (0.54–3.29)	0.53

Regression dilution *I*^2^ statistics were below 0.9 when smoking initiation was the exposure and below 0.6 for lifetime smoking (see Supplementary Table S2). Therefore, an unweighted MR Egger SIMEX correction was applied only to smoking initiation. Cochran’s *Q*-tests did provide evidence for heterogeneity (see Supplementary Table S3) but the MR Egger intercept did not differ from zero, suggesting the results were not biased by directional horizontal pleiotropy (see Supplementary Table S4). We calculated mean-F statistics to test instrument strength and all were greater than 10 (see Supplementary Table S2). We also carried out Steiger filtering to test for reverse causation. The majority of SNPs explained more variance in the exposure than in the outcome providing little evidence for reverse causation (see Supplementary Table S5). We ran our summary-level MR analysis with SNPs explaining more variance in the outcome excluded and obtained similar results (see Supplementary Table S6).

## DISCUSSION

We used multiple methods to investigate cigarette smoking as a potential risk factor for IBD and its subtypes, UC, and Crohn disease. The results of our observational analysis suggested that ever smoking increases the risk of IBD and its subtypes compared with never smoking whilst being a current vs former smoker increases the risk of Crohn disease and decreases the risk of UC, in line with previous observational literature.^2, 3^ This could suggest that exposure to tobacco smoking is a causal risk factor in the development of IBD overall, but that smoking cessation specifically decreases the risk of Crohn disease whilst increasing the risk of UC.

However, our summary-level MR results provided little evidence that smoking increases the risk of developing Crohn disease or UC particularly and, to an even lesser extent, IBD. If such an effect exists, then it is likely to be small. We found some evidence for an effect of smoking on the risk of developing UC in our individual-level MR analysis. Ever smoking and previously smoking increased the risk in line with our observational data although current smoking showed no apparent effect on risk. Conversely, our individual-level MR provided little evidence for an effect of smoking on Crohn disease or IBD.

Given the discrepancy between our observational analysis and MR analysis, it may be the case that our MR analysis was underpowered to detect an effect. This could be due to (1) a small causal effect size, (2) weak instrument bias, or (3) our sample being too small. It is unlikely that our instrument was too weak, given that we have demonstrated the mean F statistics were greater than 10 for both instruments. Furthermore, we conducted two-sample summary-level MR with large sample sizes (*N* > 59,000) suggesting a likely explanation for failure to clearly demonstrate an effect is in fact any true causal effect being smaller than that seen in observational data or even absent. The observational data may be biased by confounding factors or reverse causation, problems that we have overcome using an MR design.

In addition, we might not have found evidence for an effect of smoking because our study focused on *risk* of IBD as the outcome. Many previous studies have instead found an association between progression, symptom severity, or recurrence of IBD. For example, in Crohn disease patients, tobacco smoke is associated with worse symptoms and a worse clinical course of the disease.^5^ The biological mechanism by which this occurs remains unclear due to the chemical complexity of tobacco smoke but has been postulated, in both animal and human studies, to be due to the effects of nicotine (and perhaps other constituents of tobacco) on the immune system and microbiota of the gut.^32^ There is evidence to suggest that this could be a causal pathway, given that these effects are associated with smoking in a dose-dependent manner^33^ and that smoking cessation leads to marked improvement in disease.^34^

In the case of UC, current smoking has been associated with improved symptoms and disease course^7^ compared with former smokers. Smoking cessation meanwhile is associated with a 3-fold increased risk of being diagnosed with UC in the 5-year period following cessation.^35^ A biological mechanism has again not been clearly elucidated but there is an apparent role for nicotine with several randomized controlled trials, demonstrating that nicotine patches help to improve symptoms in active UC when compared with placebo patches.^36^ Interestingly, nicotine did not have any significant effects over placebo in the only randomized controlled trial to investigate maintenance of remission in UC,^37^ which suggests that it does not suppress risk of UC. This further supports the notion that we might not expect to see effects of cigarette smoking on IBD risk and future studies should instead focus on its role in the progression and remission of IBD diagnoses. This is currently not possible, but methods are currently being developed to extend MR to be able to do this.^38^

### Strengths and Limitations

The strengths of our study include the use of the MR design to reduce bias from reverse causation and residual confounding, as well as the many sensitivity analyses conducted to strengthen our conclusions. Our genetic instruments for smoking were not weak, demonstrated by our tests of instrument strength and both of our instruments having been validated previously.^14, 20^ We used multiple genetic instruments to capture different aspects of smoking behavior, including smoking initiation, lifetime smoking, and smoking heaviness. Our single SNP analysis allowed us to focus on a variant with known biological consequence and to include a negative control, both these factors reduce the potential for pleiotropy. However, the limitation of using a single SNP is reduced statistical power and so this alone does not allow us to conclude the absence of an effect. Our single SNP instrument also only reflects smoking heaviness and not the likelihood of smoking initiation.

As previously discussed, our results suggest that statistical power may have been a limitation of our summary-level MR analysis, especially if the true effect size is small. A strength of our study in this regard is good instrument strength and the use of the 2-sample summary-level MR design with both of our exposure samples being substantial in size. Therefore, if the study was underpowered, this probably reflects any true causal effect being small or absent.

In addition, our observational and individual-level MR samples had lower prevalence of smoking than the general population potentially reducing our power and the generalizability of these results. However, we still obtained significant results in our observational analysis and this issue was not relevant to our summary-level MR results. Conversely, a strength of our observational and individual-level MR analyses was that the prevalence of IBD in the samples used were comparable with the general population.

Finally, a limitation of MR studies is possible bias from horizontal pleiotropy (where the genetic variants affect the outcome through pathways other than through the exposure). To assess for any pleiotropy, we conducted multiple sensitivity analyses, for example, the MR Egger intercept. These tests did not suggest the presence of bias due to horizontal pleiotropy. Our Steiger directionality results also suggest that our results were not biased by reverse causation.

## CONCLUSIONS

We have not found clear evidence for an effect of smoking on the risk of developing IBD using MR analysis. Observational associations could be due to residual confounding or reverse causation. Future work should aim to explore the effects of smoking on IBD maintenance and progression rather than risk of onset.

## Supplementary Material

otaa018_suppl_Supplementary_Tables

## Notes

Funding: REW, TGR, GDS and MRM are all members of the MRC Integrative Epidemiology Unit at the University of Bristol funded by the Medical Research Council: http://www.mrc.ac.uk [MC_UU_00011/1, MC_UU_00011/7]. This study was supported by the NIHR Biomedical Research Centre at the University Hospitals Bristol NHS Foundation Trust and the University of Bristol. The views expressed in this publication are those of the authors and not necessarily those of the NHS, the National Institute for Health Research or the Department of Health and Social Care. This research has been conducted using the UK Biobank Resource under Application Number 9142.
