# Supplementary material for: Exploring the Effects of Cigarette Smoking on Inflammatory Bowel Disease Using Mendelian Randomization
Source: Crohns Colitis 360. 2020 Mar 12;2(1):otaa018. doi: 10.1093/crocol/otaa018 (PMC7809707; doi:10.1093/crocol/otaa018)
Supplement: otaa018_suppl_Supplementary_Tables [file otaa018_suppl_supplementary_tables.pdf]

Exploring the effects of cigarette smoking on inflammatory bowel disease: A Mendelian randomisation study

Contents

Table S1. Regression Dilution I^2_{GX} to test the suitability of instruments for MR Egger and the mF statistic as a test of instrument strength	**Page 3**
Table S2. Cochran's Q Tests of Heterogeneity	**Page 4**
Table S3. MR Egger Intercept test of directional horizontal pleiotropy	**Page 5**
Table S4. The number of SNPs from the genetic instrument that explain more of the variance in smoking behaviour than IBD according to Steiger directionality tests	**Page 6**
Table S5. Association of rs1051730 genotype with smoking behaviours and demographic factors in a sample of 337,053 individuals from the UK Biobank	**Page 7**
Supplementary Table 6. Two-sample Mendelian randomisation analysis of the effect of genetic predisposition to smoking behaviours on risk of IBD and its subtypes using only SNPs that explain more variance in exposure than outcome	**Page 8**

Supplementary Table S1. Regression Dilution I^2_{GX} to test the suitability of instruments for MR Egger and the mF statistic as a test of instrument strength

Exposure	Outcome	mF	I^2 Weighted G_X	I^2 Unweighted G_X
**Lifetime Smoking**	IBD	44.05	0.407	0.632
	Crohn's Disease	44.05	0.406	0.632
	Ulcerative Colitis	44.05	0.410	0.632
**Smoking Initiation**	IBD	44.89	0.393	0.613
	Crohn's Disease	44.88	0.392	0.614
	Ulcerative Colitis	44.82	0.400	0.620

Supplementary Table S2. Cochran's Q Tests of Heterogeneity

Exposure	Outcome	IVW Q (Degrees of freedom)	IVQ Q P-value
**Lifetime Smoking**	IBD	271.63 (106)	<0.001
	Crohn's Disease	220.02 (106)	<0.001
	Ulcerative Colitis	185.49 (106)	<0.001
**Smoking Initiation**	IBD	700.16 (324)	<0.001
	Crohn's Disease	612.26 (324)	<0.001
	Ulcerative Colitis	544.20 (324)	<0.001

Supplementary Table S3. MR Egger Intercept test of directional horizontal pleiotropy

Exposure	Outcome	MR Egger Intercept	Standard Error	P-value
**Lifetime Smoking**	IBD	0.00617	0.00865	0.48
	Crohn's Disease	0.0122	0.0100	0.22
	Ulcerative Colitis	0.00108	0.00909	0.91
**Smoking Initiation**	IBD	0.00504	0.00471	0.29
	Crohn's Disease	0.00576	0.00569	0.31
	Ulcerative Colitis	0.00486	0.00528	0.36

Supplementary Table S4. The number of SNPs from the genetic instrument that explain more of the variance in smoking behaviour than IBD according to Steiger directionality tests

Exposure	Outcome	True (%)	False
Lifetime Smoking	IBD	115 (92.0)	10
	Crohn’s disease	108 (86.4)	17
	Ulcerative Colitis	112 (89.6)	13
Smoking Initiation	IBD	357 (95.5)	17
	Crohn’s disease	352 (94.4)	21
	Ulcerative Colitis	354 (94.6)	20

Supplementary Table 5. Association of rs1051730 genotype with smoking behaviours and demographic factors in a sample of 337,053 individuals from the UK Biobank

	Never smokers				Ever smokers			
	0	1	2	p-value	0	1	2	p-value
Study sample (n)	82,130	81,580	20,334		68,562	66,953	16,307	
Cigarettes per day					14.88	15.89	16.72	<0.001
Smoking status (%)[a]								
Current					22	22	22	
Former					78	78	78	0.057
Men (%)	42	41	41	0.121	52	52	52	0.539
Age (mean)	56.1	56.2	56.2	0.092	57.8	57.7	57.6	0.002
Education (%)								
Primary	14	14	14		21	21	20	
Secondary	51	50	50		51	52	52	
Tertiary	36	36	36	0.776	27	27	28	0.020
Missing (n)	683	704	202		634	685	133	
Alcohol intake (%)								
Never	7	7	7		5	6	6	
Special occasions	11	12	11		9	9	9	
Monthly	12	13	12		9	10	10	
Weekly	53	52	53		49	48	47	
Daily/almost daily	16	16	16	0.161	28	28	28	0.074
Missing (n)	33	47	16		47	55	13	
Diagnosis of IBD (%)	0.92	0.86	0.82	0.270	1.26	1.42	1.31	0.040
Diagnosis of UC (%)	0.66	0.60	0.59	0.243	0.86	1.01	1.00	0.014

Diagnosis of Crohns (%)	0.34	0.34	0.30	0.635	0.51	0.52	0.42	0.265

Supplementary Table 6. Two-sample Mendelian randomisation analysis of the effect of genetic predisposition to smoking behaviours on risk of IBD and its subtypes using only SNPs that explain more variance in exposure than outcome

Exposure	Outcome	MR Method	OR (95% CI)	P-value
**Smoking Initiation**	**IBD**	Inverse Variance Weighted	1.03 (0.93-1.13)	0.60
		MR Egger	0.91 (0.60-1.37)	0.65
		Weighted Median	1.03 (0.91-1.18)	0.61
		Weighted Mode	0.99 (0.70-1.41)	0.96
	**Crohn's Disease**	Inverse Variance Weighted	1.08 (0.96-1.21)	0.21
		MR Egger	0.74 (0.45-1.21)	0.23
		Weighted Median	1.07 (0.91-1.26)	0.41
		Weighted Mode	1.20 (0.71-2.02)	0.50
	**UC**	Inverse Variance Weighted	0.99 (0.88-1.11)	0.85
		MR Egger	0.85 (0.54-1.34)	0.48
		Weighted Median	1.08 (0.93-1.27)	0.31
		Weighted Mode	1.17 (0.71-1.94)	0.53
**Lifetime Smoking**	**IBD**	Inverse Variance Weighted	1.01 (0.80-1.27)	0.94
		MR Egger	0.58 (0.23-1.42)	0.23
		Weighted Median	1.01 (0.76-1.36)	0.93
		Weighted Mode	1.10 (0.59-2.03)	0.77
	**Crohn's Disease**	Inverse Variance Weighted	1.13 (0.87-1.46)	0.36
		MR Egger	0.68 (0.25-1.83)	0.45
		Weighted Median	1.36 (0.94-1.97)	0.10
		Weighted Mode	1.60 (0.63-4.05)	0.32

	**Ulcerative Colitis**	Inverse Variance Weighted	0.90 (0.70-1.15)	0.41
		MR Egger	1.17 (0.44-3.14)	0.75
		Weighted Median	0.98 (0.69-1.40)	0.91
		Weighted Mode	1.59 (0.62-4.12)	0.34
